# Food Availability and Maternal Immunization Affect Transfer and Persistence of Maternal Antibodies in Nestling Pigeons 

**DOI:** 10.1371/journal.pone.0079942

**Published:** 2013-11-05

**Authors:** Ahmad Ismail, Lisa Jacquin, Claudy Haussy, Julie Legoupi, Samuel Perret, Julien Gasparini

**Affiliations:** 1 Laboratoire Ecologie and Evolution UMR 7625, Université Pierre et Marie Curie CNRS ENS, Paris, France; 2 Department of Biology and Redpath Museum, McGill University, Montréal, Québec, Canada; 3 Centre d’Ecologie Expérimentale et Prédictive CEREEP-Ecotron Ile-De-France CNRS ENS, UMS 3194, Ecole Normale Supérieure, St-Pierre-lès-Nemours, France; University of Jyväskylä, Finland

## Abstract

The ability of mothers to transfer antibodies (Abs) to their young and the temporal persistence of maternal Abs in offspring constitute important life-history traits that can impact the evolution of host-parasite interactions. Here, we examined the effects of food availability and parental immunization on the transfer and persistence of maternal antibodies in nestling pigeons (*Columba livia*). This species can transmit maternal Abs to offspring before hatching through the egg yolk and potentially after hatching through crop milk. However, the role of this postnatal substance in immunity remains elusive. We used a full cross-fostering design to disentangle the effects of food limitation and parental immunization both before and after hatching on the levels and persistence of maternal Abs in chicks. Parents were immunized via injection with keyhole limpet hemocyanin antigens. Using an immunoassay that specifically detected the IgY antibodies that are known to be transmitted via the yolk, we found that the levels of anti-KLH Abs in newly hatched chicks were positively correlated with the levels of anti-KLH Abs in the blood of their biological mothers. However, this correlation was not present between chicks and their foster parents, suggesting limited IgY transfer via crop milk to the chick’s bloodstream. Interestingly, biological mothers subjected to food limitation during egg laying transferred significantly fewer specific maternal Abs, which suggests that the transfer of antibodies might be costly for them. In addition, the persistence of maternal Abs in a chick’s bloodstream was not affected by food limitation or the foster parents’ anti-KLH Ab levels; it was only affected by the initial level of maternal anti-KLH Abs that were present in newly hatched chicks. These results suggest that the maternal transfer of Abs could be costly but that their persistence in an offspring’s bloodstream may not necessarily be affected by environmental conditions.

## Introduction

The immune systems of newly born offspring are not entirely mature and therefore cannot provide complete protection when pathogens are first encountered in the external environment [1]. The transfer of maternal antibodies (Abs) may thus have evolved as a way to reduce the negative impact of pathogens on immature juveniles [2]. This transgenerational source of immunological plasticity has received growing attention as it is a widespread phenomenon with broad implications [1,3,4]. In vertebrates, maternal Abs can be transferred to young either before birth via the egg yolk or the placenta and/or after birth via the colostrum and milk [1]. Maternal Abs have been shown to affect juvenile immunity at different time scales [5,6] (but see 7,8), directly participate in a juvenile’s immune response to antigens [9–11], impact fitness [3,4] (but see 7), and potentially influence the dynamics of host-parasite interactions [12]. The transfer of maternal Abs might thus be a crucial life-history trait that can shape the evolution of host-parasite interactions at different evolutionary time scales [5–7].

Individuals vary greatly in the amount of maternal Abs they transfer to their offspring, but the evolutionary forces shaping this variability are still poorly characterized [13]. In this study, we focused on two types of variation associated with this important life-history trait: 1) the initial amount of maternal Abs transferred prenatally, and 2) Ab persistence in the offspring’s bloodstream after hatching. Indeed, previous work suggests that females with similar levels of Abs in their bloodstreams may nonetheless differ in their ability to transfer those Abs [13]. Additionally, the temporal persistence of maternal Abs in the offspring’s bloodstream has recently been shown to be highly variable among species, with a higher level of persistence being associated with a longer period of time during which protection may be conferred to nestlings [14]. Maternal Ab persistence, as well as the rate of initial transfer, might thus be under selective pressure. The temporal persistence of Abs in an offspring’s bloodstream is known to be affected by the initial amount of maternal Abs it receives [15], which is itself directly linked to the Ab level in maternal blood [16]. Furthermore, because offspring have the ability to catabolize maternal Abs and thus affect their temporal persistence, environmental conditions that affect physiological processes are likely to play an important role in offspring immunity. As a consequence, the persistence of maternal antibodies might be shaped by selective forces acting on both parental and offspring phenotypes, while the ability of mothers to transfer Abs might be shaped by selective forces acting on adult females alone [3]. However, little is known about the factors affecting the ability to transfer maternal Abs, the temporal persistence of maternal Abs in juveniles, and the energetic costs for females and offspring. 

Our study focused on two environmental factors that may affect maternal Ab transfer and persistence: variation in food availability and parental exposure to parasites. If transferring Abs is costly, mothers in poor nutritional condition may be less capable of transferring maternal Abs to their offspring. Similarly, Abs may persist for shorter time periods in juveniles in poor nutritional condition if Ab maintenance incurs costs. In contrast, if poor food availability means that future residual reproductive value is diminished, individuals in poorer condition may invest more heavily in current reproductive efforts and thus transfer larger amounts of maternal Abs that will persist longer. Support for this idea comes from a study in which kittiwake (*Rissa tridactyla*) mothers transferred higher levels of maternal Abs into their eggs when their food intake was experimentally reduced [17]. Likewise, in the Indian meal moth (*Plodia interpunctella*), mothers in poor nutritional condition increased the transfer of maternal resistance to juveniles [18]. In contrast, in the Japanese quail (*Coturnix japonica*), protein manipulation had no effect on the transfer of maternal Abs into eggs [19]. However, nothing is known about the effect of food availability on the persistence of maternal Abs in juveniles. We alternatively expect that increased food intake could increase the ability of young to catabolize maternal Abs and may thus decrease the persistence of maternal Abs. Also, young in better condition may show higher growth rates, which could dilute their maternal Ab levels and thus decrease their persistence.

In bird species that are able to transmit postnatal immune substances through crop milk, postnatal parental exposure to parasites may increase the level of parental Abs transferred via crop milk, which, in turn, could contribute to the persistence of parental Abs in the offspring’s blood. Indeed, in many mammal species (such as rodents) postnatal Abs enter neonatal blood circulation after being transported across the intestinal epithelium [20]. In human**s**, in contrast, colostrum and milk are mainly composed of immunoglobulins A (IgAs), which are believed to play a central role in protecting the gut against local infections but do not cross the intestinal epithelium to reach neonatal circulation [20]. It is therefore possible that parental Abs transferred after birth by those bird species able to produce crop milk may significantly increase the levels of maternal Abs circulating in juveniles and prolong Ab presence as juveniles age, a phenomenon that is known to occur in certain mammal species [21,22].

To test these hypotheses, we used a cross-fostering experiment to test the effect of food supplementation and parental immunization on Ab transfer and persistence in feral pigeons (*Columbia livia*). These columbid birds feed their chicks crop milk [23], a lipid-rich substance produced in their crops that is known to contain nutritive and growth factors [24] as well as carotenoids [25] and immunoglobulins [26], and that may have a role in offspring immunity [6]. This bird model thus offers us a unique opportunity to investigate the relative effects of pre- and postnatal variation in food availability and parental immunization on initial Ab levels in offspring blood and the persistence of maternal Abs over time. We have previously shown that parental immunization before egg laying can affect the juvenile immune response after hatching and the total immunoglobulins present in crop milk in this species [6]. However, it is still unclear how environmental factors such as prenatal and postnatal food availability and parental immunization interact to shape Ab transfer amounts and Ab persistence in young.

## Materials and Methods

### Study populations

A total of 120 adult feral pigeons (60 females and 60 males) were captured with trap cages at three suburban locations near Paris in 2010 by the SACPA Company (France) with the agreement of local authorities (no specific permission was required). They were kept in 10 outdoor aviaries (2.20 m x 2.20 m) at the CEREEP field station (CEREEP-Ecotron Ile-de-France, UMS 3194, Ecole Normale Supérieure, St-Pierre-les-Nemours). Each aviary contained six males and six females. On the day of clutch completion, the two eggs of each clutch were cross-fostered with another nest with a similar laying date (± 1 day) to create experimental groups of chicks differing in the prenatal and postnatal parental immunization and food availability treatments experienced by biological and foster parents. 

### Food treatment

The food limitation treatment was initiated two weeks before the immunization treatment. Sixty pigeons (in five aviaries) were food limited (“food-limited group”): they were fed 30 g of wheat per day per individual, which corresponds to the basal food quantity used to maintain non-breeding pigeons [27]. When the chicks in this group hatched, no food was added during their first week of life. During their second week of life, 15 g per chick per day were added. From the third week onwards, 30 g per chick per day were added. Sixty other pigeons (in five aviaries) were fed a mixture of corn, wheat, and peas *ad libitum* (“*ad libitum* food group”). All pigeons were provided with mineral grit and vitamin-supplemented water. Such food regimes have previously been shown to significantly affect reproduction and condition in adult feral pigeons [28].

### Parental and chick immunization treatment

The immunization treatment started two weeks after the beginning of the food treatment. The antigen-injected group consisted of 60 birds (three breeding pairs chosen randomly in each aviary) that received a subcutaneous injection of a 100-μL solution containing 0.5 mg.mL^-1^ keyhole limpet hemocyanin (KLH). KLH is a natural protein that birds do not encounter in their natural environment, and it is therefore used to stimulate an immune response to a novel antigen [8,29,30]. It is a copper-containing respiratory protein derived from the keyhole limpet (*Megathura crenulata*) [31]. It stimulates a strong Ab response but does not trigger a severe inflammatory or pyrogenic response as do many other immunogens [32]. The sham-injected group consisted of the 60 remaining birds that were injected with phosphate-buffered saline (PBS). A second injection was performed two weeks later to ensure that blood anti-KLH Ab levels differed between antigen- and sham-injected treatment groups. Then, we monitored the nest**s** daily in order to record laying and hatching dates. Females were captured twice to collect blood samples, once at the time of egg laying (1.6 ± 0.5 days after egg laying) and once at the time of hatching (3.1 ± 0.5 days before hatching). Blood was taken from the brachial vein using a sterile syringe, and the sample was used to assess anti-KLH Ab levels in adult blood at the time of egg laying and hatching. As fathers also fed offspring with crop milk and were thus possibly transferring Abs, we collected blood samples from males to assess their anti-KLH Ab levels at the time of hatching. To examine the persistence of parental anti-KLH Abs in offspring, we collected blood samples from chicks as they aged at 3, 7, 14, and 21 days post-hatching. All blood samples were centrifuged, and the plasma was stored at -20°C until immunological assays could be conducted. It should be noted that clutches were laid 64.46 ± 2.23 days after the beginning of the food treatment and 50.46 ± 2.23 days after the beginning of the immunization treatment. These intervals did not differ significantly between food and immunization treatment groups (all P-values > 0.05).

### Anti-KLH antibody assay

Anti-KLH IgY Ab levels in the plasma samples were assayed using the method described in [13]. High-binding plates (96 wells, flat bottom, Microlon® 600; cat. 655101, Greiner Bio-One, Germany) were coated overnight at 5°C with 100 μL of KLH (40 μg.mL^-1^ in 50 mM carbonate/bicarbonate buffer, pH 9.6) and then washed five times with PBS (0.1 M, pH 7.4). Wells were blocked with 200 μL of a 3% milk powder-PBS solution (dried milk obtained from Régilait Bio) for two hours at room temperature with agitation. After five washings, 100 μL of plasma (dilution 1/500) diluted in a 0.5% milk powder-PBS solution were added to the wells. Plates were then incubated overnight at 4°C. After washing, 100 μL of rabbit-anti-pigeon IgG conjugated to horseradish peroxidase (10 mg/ml in PBS, pH 7.2, Nordic Immunology, Netherlands) and diluted in a 0.5% milk powder-PBS solution (dilution 1/5000) were added to the wells; the plates were then incubated for two hours at room temperature. After washing, 100 μL of ortho-phenylenediamine (OPD) (Sigma-Aldrich, USA) were added to the wells. The reaction was stopped after 10 minutes by adding 50 μL of HCl (1 M). Plates were then read at 490 nm using a microplate reader (Model 680, Bio-Rad Laboratories, UK). A panel of samples from another experiment was used to estimate the repeatability [33] of sample Ab values between plates (N = 56, F_53,56_ = 55.8, P < 0.001, r = 0.96) as well as within plates (N = 31, F_26,31_ = 21.3, P < 0.001, r = 0.89); the results showed that this method reliably measures anti-KLH Ab levels. A mixture of several pigeon samples was serially diluted and added to all plates to standardize the relative Ab concentrations of all the samples. The relative concentration of anti-KLH Abs in the samples was log transformed and is referred to hereafter as the anti-KLH Ab level. 

### Ethics statements

This study was carried out in strict accordance with the recommendations of the European Convention for the Protection of Vertebrate Animals used for Experimental and Other Scientific Purposes (revised Appendix A). All experiments were approved by the “Direction Départementale des Services Vétérinaires de Seine-et-Marne” (authorization No. 77-05). This study does not involve endangered or protected species.

### Statistical analyses

We obtained blood samples at four different ages for 58 chicks. These chicks came from the 31 biological nests and 29 foster nests for which anti-KLH Ab levels of biological and foster parents were available. To test whether the initial Ab level in newly hatched chicks was affected by food limitation, parental immunization, and parental Ab levels, we performed a generalized mixed model in which the Ab level of 3-day-old chicks was the response variable, the food treatment group of the biological parents was an explanatory variable, and the Ab levels of the biological mothers, foster mothers, and foster fathers were covariates. As sibling chicks are not statistically independent, we included the biological nest, nested within the food treatment group of the biological parents, as a random factor. To test whether the food treatment group and Ab levels of foster parents affected maternal Ab persistence in chicks, we performed a generalized mixed model in which chick anti-KLH Ab level across time was the response variable, the food treatment group of the foster parents and chick age (7, 14, and 21 days old) were explanatory variables, and the anti-KLH Ab levels of 3-day-old chicks (i.e. initial amount of anti-KLH Abs received from biological mothers) and the anti-KLH Ab levels of foster fathers and mothers were covariates. Foster nest, nested within the food treatment group of the foster parents, was included as a random factor. The best-fitting models were chosen using the AIC criterion. All statistical analyses were performed using SAS (version 9.2).

## Results

**Figure 1:**
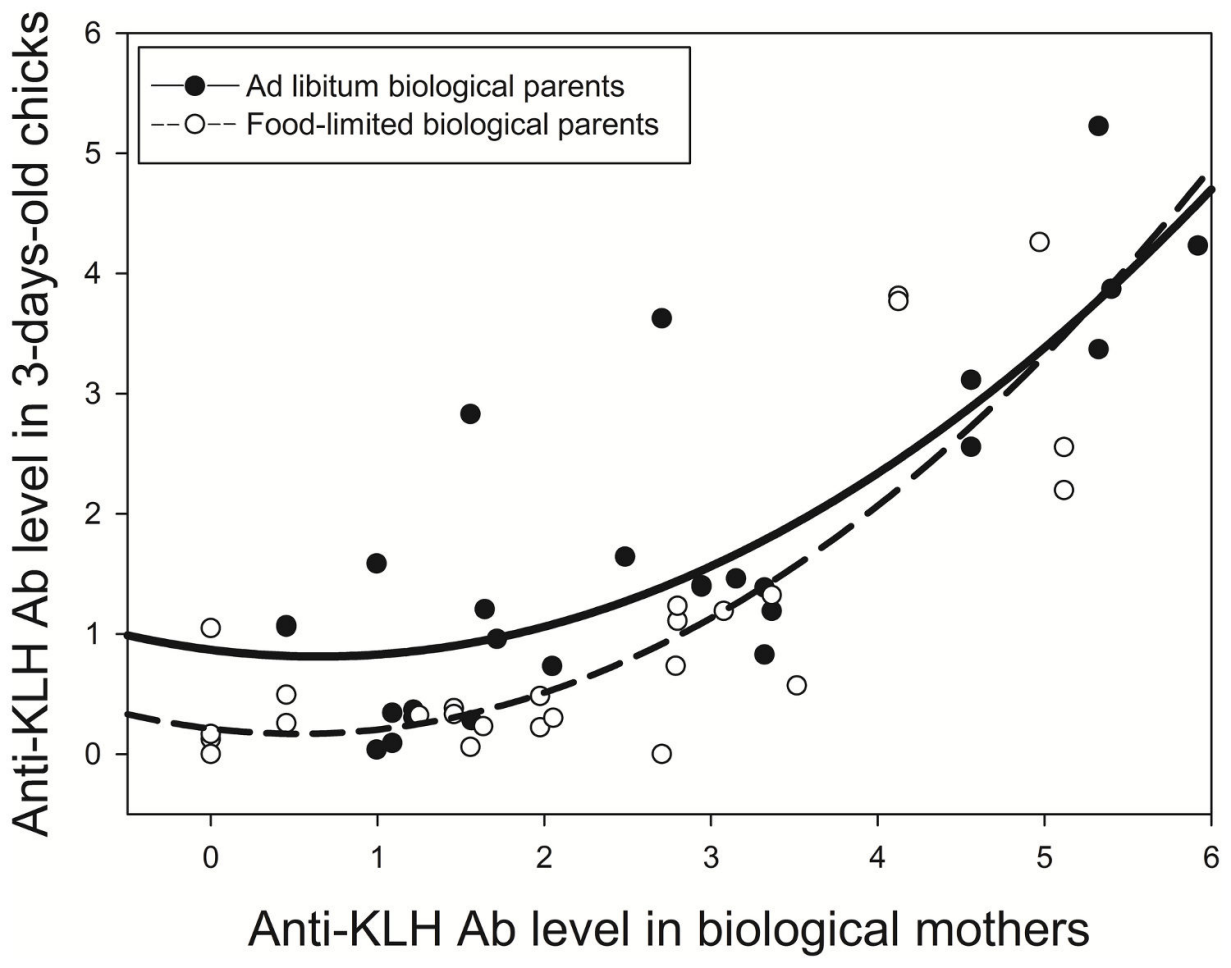
Positive quadratic relationships between anti-KLH Ab levels in 3-day-old chicks and their biological mothers. Dot color indicates food treatment (white = food-limited; black = *ad* *libitum*).

KLH-injected adults had higher anti-KLH Ab levels than sham-injected adults at the time of egg laying in the case of biological mothers (*Student’s t-test*, t_30_ = 6.24, p < 0.0001) and at the time of hatching in the case of foster parents (mothers and fathers pooled: t_57_ = 3.08, p = 0.003), which shows that antigen injection triggered a significant humoral immune response in parents. We found a positive, quadratic relationship between anti-KLH Ab levels in biological mothers and those in 3-day-old chicks (Table 1, Figure 1), which provides evidence that the amount of Abs transferred to offspring was a function of the amount circulating in the mother’s blood at laying. In addition, for a given level of circulating maternal Abs, 3-day-old chicks from food-limited biological mothers had lower Ab levels than chicks from *ad libitum* biological mothers (Table 1, Figure 1). There was no relationship between the Ab levels of foster mothers and fathers and the Ab levels of 3-day-old chicks (Table 1).

**Table 1:** Output of the best-fit generalized mixed model that explains anti-KLH Ab levels in 3-day-old chicks.

**Effects**	**DF**	**F**	**P-values**
Anti-KLH Ab level of the biological mother	1,19	2.29	0.15
(Anti-KLH Ab level of the biological mother)²	1,19	19.10	0.0003
Food treatment experienced by the biological mother	1,30	5.40	0.03

The quadratic anti-KLH Ab level of the biological mother and the food treatment experienced by the biological mother were significant main effects in this model. All models involving interactions or the anti-KLH Ab levels of the foster parents had higher AICc values (>2) than the model presented here.

**Figure 2:**
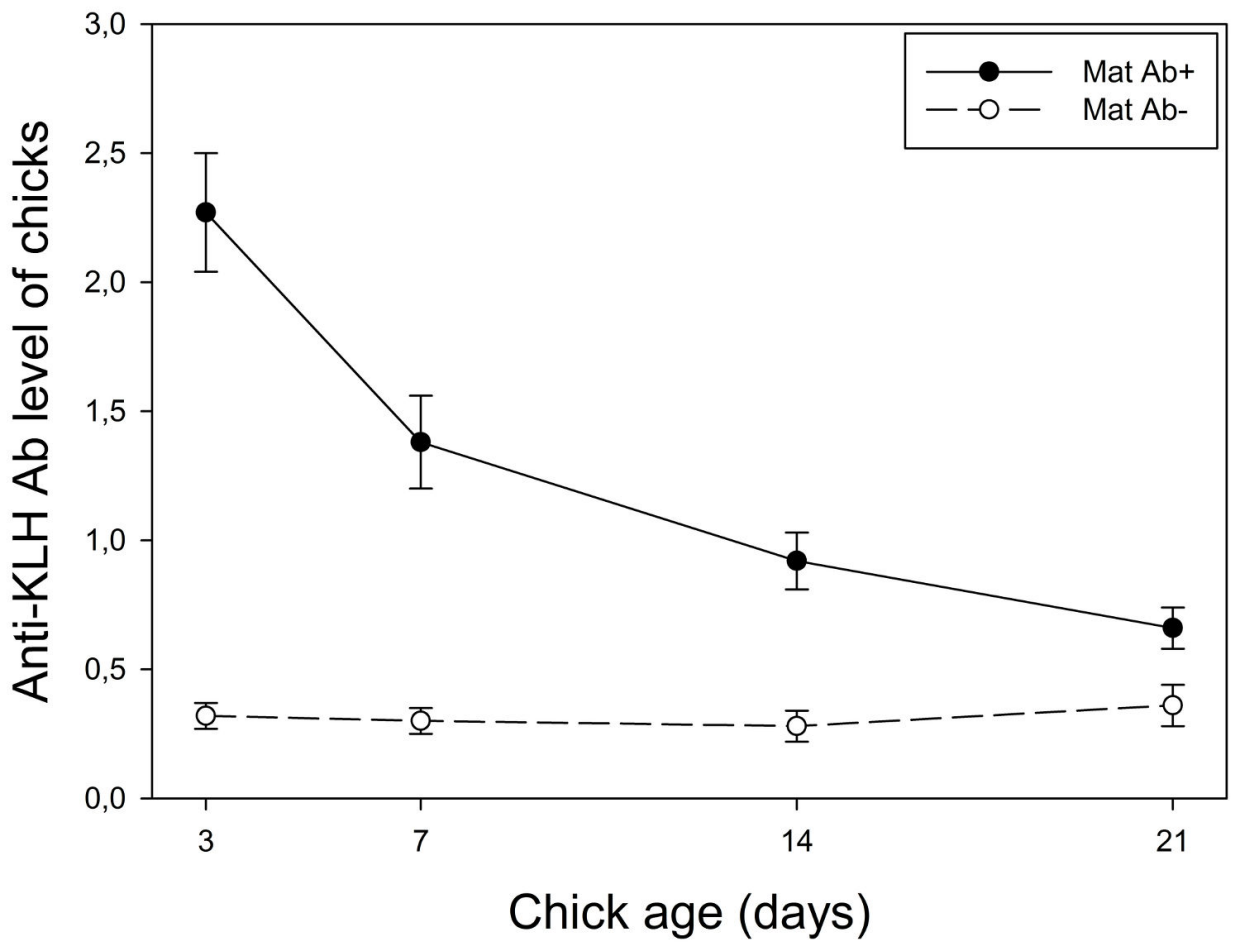
Persistence of maternal anti-KLH Abs in chicks as they age. For illustrative purposes, we split the chicks into two categories on the basis of the median 3-day-old anti-KLH Ab level: chicks that have received high levels of maternal anti-KLH Abs (Maternal Ab+; above the median) are represented by black dots and chicks that have received low levels of maternal anti-KLH Abs (Maternal Ab-; below the median) are represented by white dots.

**Figure 3:**
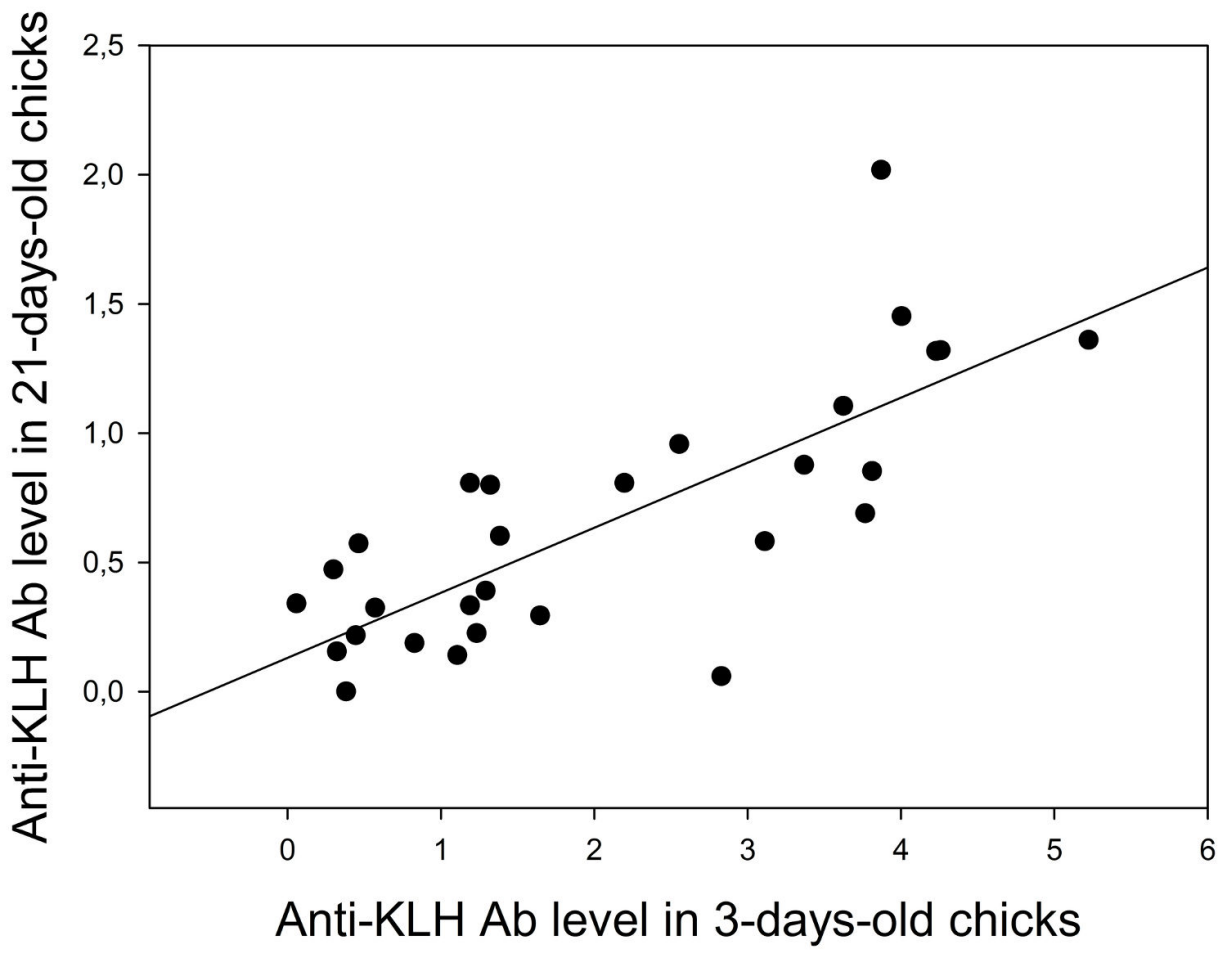
Positive relationship between anti-KLH Ab levels in 3- and 21-day-old chicks from immunized biological mothers.

The persistence of maternal anti-KLH Abs was only affected by the initial level of maternal Abs found in 3-day-old chicks (Table 2, Figure 2). Chicks with higher anti-KLH Ab levels at 3 days of age (corresponding to those coming from immunized biological mothers; see Figure 1) showed a stronger decline in anti-KLH Ab levels over time but those levels were nonetheless higher at 7, 14, and 21 days of age than those in chicks coming from non-immunized biological mothers (Figure 2). Accordingly, in chicks coming from immunized biological mothers, there were significant positive correlations between chick anti-KLH Ab levels at 3 and 7 days (N = 30, r = 0.95, p < 0.0001), 3 and 14 days (N = 30, r = 0.85, p < 0.0001), and 3 and 21 days (N = 30, r = 0.78, p < 0.0001, Figure 3). Neither the anti-KLH Ab levels nor the food treatment experienced by foster parents affected the persistence of anti-KLH Abs in chicks (main factor or interaction; see Table 2).

**Table 2:** Output of the best-fit generalized mixed model that explains variation in anti-KLH Ab levels in chicks as they age.

**Effects**	**DF**	**F**	**P-values**
Initial anti-KLH Ab level at 3 days old	1,113	303.52	< 0.0001
Chick age (7, 14, and 21 days)	2,113	4.30	0.03
Chick age x Initial anti-KLH Ab level at 3 days old	2,113	51.82	< 0.0001

The interaction between chick age and initial anti-KLH Ab levels in 3-day-old chicks was significant in this model. All models involving other interactions, the food treatment, or the anti-KLH Ab levels of foster parents had higher AICc values (>2) than the model presented here.

## Discussion

In this study, we first tested if food availability during egg laying and the immunization of foster parents affected the maternal transfer of specific Abs to young chicks. As previously reported [16], we found that the level of specific anti-KLH Abs in young chicks was positively correlated with that found in maternal blood during egg laying (Figure 1). Interestingly, the shape of this correlation was quadratic, suggesting that the rate of transfer increases as the level of anti-KLH Abs circulating in mothers increases. Contrary to our prediction, the level of anti-KLH Abs in 3-day-old chicks was not affected by the level circulating in foster parents (mothers and/or fathers). This result suggests either that parental crop milk did not contain Abs or that such Abs did not cross the chick gut barrier to reach the bloodstream. Previous studies have quantified total Abs in the crop milk of pigeons [26]. The crop milk of columbids contains mostly IgAs that are believed to play a local protective role in the gut of young nestlings and only partially reach the bloodstream [26]. In this study, we measured levels of specific anti-KLH IgYs, not IgAs, in young, which may explain why the immunization of foster parents did not affect offspring Ab levels. Another recent study also failed to detect anti-KLH IgYs in pigeon crop milk following immunization [6]. However, the study also found that a parental immune challenge affected the long-term humoral response of one-year-old juveniles [6]. This finding suggests that postnatal transfer of immunity occurred by means other than IgG transfer (likely IgA transfer) and affected the long-term immune responses of nestlings. The next step to validate this hypothetical mechanism would be to quantify IgAs in crop milk and the chick bloodstream. 

Controlling for anti-KLH levels in biological mothers, we found that the food treatment experienced during egg laying significantly affected maternal Ab transfer (Table 1); specifically, food limitation during egg laying significantly decreased the amount of Abs transferred. In contrast to previous studies [17,19], food limitation appears here to be an important factor that decreases the maternal transfer of Abs, a crucial life-history trait [1]. This discrepancy may be explained by the fact that we examined specific Abs (anti-KLH) in our study, whereas previous studies focused exclusively on total Abs. Measuring total Abs can yield a different result since both induced and non-induced Abs may be present [19,34,35]. Our study suggests that the transfer of induced Abs might be costly for females, an issue that is still debated. This cost might stem from the accumulation of high levels of maternal antibodies in eggs, which may constitute a significant resource drain for ovulating females [1,36]. As a consequence, trade-offs may occur with other life-history traits and strategic adjustments may have evolved to cope with different environmental factors such as pathogen exposure and food availability, which would explain variation among females in Ab transmission ability. The effect of food availability on the transfer of specific maternal Abs identified in this study could have important consequences for the ecology and evolution of host-parasite interactions. Further experiments involving the manipulation of exposure to natural antigens are now needed to investigate the trade-offs between maternal transfer ability and other life-history traits [37].

 The second objective of this study was to examine the temporal persistence of maternal anti-KLH Abs in growing chicks, which is an oft-neglected life-history trait [14]. To this end, we examined the variation in anti-KLH Ab levels in chicks as they aged (at 3, 7, 14, and 21 days). First, we found that Ab level decreased strongly as a function of nestling age in chicks that had received anti-KLH Abs from immunized parents (Figure 2). As previously shown in the Japanese quail [15], the duration of Ab persistence is predicted by the initial level of maternal anti-KLH Abs received by chicks, as shown by the positive relationship found between anti-KLH Ab levels at 3 and 21 days (Figure 3). As previously discussed, the temporal persistence of Abs in nestlings was not affected by the hypothetical transfer of postnatal Abs by foster parents. In addition, we did not find any effect of the food treatment experienced during growth on this persistence, suggesting that it is not energetically costly to keep maternal Abs for offspring and that food intake does not increase the ability of young to catabolize maternal Abs. Recent studies have underscored the importance of maternal Ab persistence and have shown that it demonstrates interspecific variation that may be linked to species longevity [14]. For instance, maternal Abs persist longer in the longer-lived Cory’s shearwater (*Calonectris diomedea*) than in the shorter-lived kittiwake (*Rissa tridactyla*) [14,16]. Our study further emphasizes that studies on maternal Ab persistence should take into account the initial level of Abs transferred by mothers. 

 In conclusion, our experimental study shows that although food availability can affect the ability of mothers to transfer Abs via the egg yolk to their chicks, it did not have a detectable effect on the duration of Ab persistence in young chicks. Furthermore, we did not detect a postnatal transfer of circulating IgY Abs by foster parents to the chick bloodstream through crop milk. Our study suggests that Ab transfer could be costly for mothers and that food availability may contribute to variation in the maternal ability to transfer Abs to young. Taken together, these results suggest that the prenatal environment might have a strong effect on an offspring’s circulating Ab levels via the prenatal transfer of Abs by biological mothers, whereas postnatal conditions might have less of an effect on this trait. More studies are required to better understand variation in the transfer and temporal persistence of Abs among individuals and/or species.
